# Correction to ‘EMPIAR: The Electron Microscopy Public Image Archive’

**DOI:** 10.1093/nar/gkad201

**Published:** 2023-03-27

*Nucleic Acids Research*, Volume 51, Issue D1, 6 January 2023, Pages D1503–D1511, https://doi.org/10.1093/nar/gkac1062

In the originally published version of this manuscript, there was an error in the scaling shown in Figure 2 – these should be in terabytes and not in gigabytes.


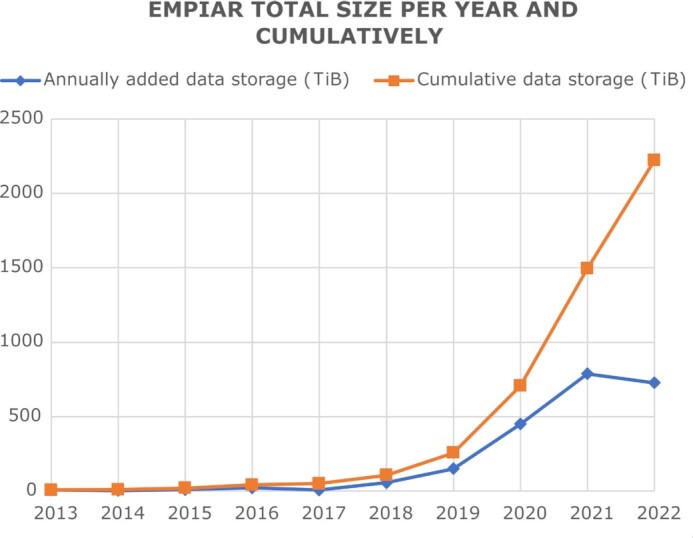


This error has been corrected in Figure 2 of the article.

